# Angiographical Identification of Intracranial, Atherosclerosis-Related, Large Vessel Occlusion in Endovascular Treatment

**DOI:** 10.3389/fneur.2019.00298

**Published:** 2019-04-16

**Authors:** Jang-Hyun Baek, Byung Moon Kim

**Affiliations:** ^1^Department of Neurology, Kangbuk Samsung Hospital, Sungkyunkwan University School of Medicine, Seoul, South Korea; ^2^Department of Radiology, Interventional Neuroradiology, Severance Stroke Center, Severance Hospital, Yonsei University College of Medicine, Seoul, South Korea

**Keywords:** intracranial atherosclerosis, angiography, occlusion type, endovascular treatment, acute stroke

## Abstract

Identification of intracranial, atherosclerosis-related, large vessel occlusion (ICAS-LVO) is important to set up an optimal endovascular treatment strategy, as most ICAS-LVOs require specific endovascular modalities for efficient recanalization. However, there is currently no decisive way to identify ICAS-LVO for endovascular treatment. Instead of the few, non-specific, clinical and imaging findings that operators have depended on, this review focused on the occlusion type, one of angiographical methods to identify the ICAS-LVO. Occlusion type was originally devised for predicting procedural details and endovascular outcomes of ICAS-LVO. Among occlusion types, truncal-type occlusion is regarded as a surrogate marker for ICAS-LVO. Although rare, false positives or negatives in truncal-type occlusion are possible. Nonetheless, occlusion type was easy to apply and reliably predictive of procedural outcomes. Furthermore, occlusion type can be determined prior to the procedure, which could allow it to be more helpful in setting up an optimal strategy before starting endovascular treatment.

## Introduction

Mechanical thrombectomy has become a standard treatment for acute, intracranial, large vessel occlusion (LVO) (1–3). Clinical outcomes of patients with an intracranial LVO have been remarkably improved by mechanical thrombectomy, and an improved recanalization rate was one of most important factors for these favorable outcomes (4). With modern endovascular devices (e.g., stent retriever, contact aspiration thrombectomy), 70–90% successful recanalization rates have been reported (5–9). Nevertheless, there are still a few problems with these devices. In spite of high recanalization rates, 40% futile recanalization rates—patients whose functional status is not independent—have been reported after endovascular treatment (EVT) (3, 10, 11). This might be because patient outcome is also affected by many other clinical and procedural factors, including time to recanalization, system of stroke care, and post-procedural management (12–14). In this regard, one of the most important modifiable factors is to set up an optimal endovascular strategy, because LVO can be effectively recanalized within a shorter timeframe using the optimal endovascular strategy (9).

Acute, intracranial, atherosclerosis-related LVO (ICAS-LVO) is not rare (15). The reported frequency of ICAS-LVO in EVT-eligible patients varies across studies, ranging from 5 to 36% (16–28). More exactly, the frequency of ICAS-LVO depends on the definition used and the patients' ethnicities, locations of occlusions, and eligible criteria for EVT (Table 1). In several studies from Korea, ICAS-LVO was found in 12–30% of study patients. Under the definition of significant fixed focal stenosis (FFS), the frequency was about 15–20%, although the precise definition was slightly variable across studies. Occlusion type, one of the major definitions used for ICAS-LVOs, also showed a similar range of frequencies, at about 12–18% (17, 18). ICAS-LVO is known to be more frequent in the posterior circulation (24, 32). In fact, one study reported that about 37% of ICAS-LVOs were in the posterior circulation (22). Based on angiographical determination, ICAS-LVOs seem to be less frequent in Western studies, at about 5–8% of EVT-eligible patients (19, 29).

**Table 1:** Frequencies of intracranial atherosclerosis-related large vessel occlusion (ICAS-LVO) in endovascular treatment-eligible patients.

**Study**	**Definition of ICAS-LVO**	**Nation of study population**	**Anterior or posterior circulation**	**Total number of patients included**	**ICAS-LVO%**
Matias-Guiu et al. (29)	TOAST classification	Spain	Both	88	17.0
Kang et al. (30)	Fixed focal stenosis^a^	Korea	Both	132	30.3
Gascou et al. (19)	Intracranial stenosis	France	Both	144^b^	5.5
Al Kasab et al. (16)	Significant fixed focal stenosis	US	Both	435	8.3
Lee et al. (24)	Significant fixed focal stenosis	Korea	Both	158	15.2
Lee et al. (23)	Significant fixed focal stenosis	Korea	Both	53^c^	17.0
Yi et al. (26)	Significant fixed focal stenosis	China	Both	55	21.8
Yoon et al. (28)	Significant fixed focal stenosis	Korea	Both	172	22.9
Jia et al. (20)	Significant fixed focal stenosis	China	Anterior	140	34.0
Kim et al. (22)	Significant fixed focal stenosis	Korea	Posterior	51^d^	37.3
Baek et al. (31)	Truncal-type occlusion (by DSA)	Korea	Both	259	12.4
Baek et al. (18)	Truncal-type occlusion (by CTA)	Korea	Both	238	18.1

a *ICAS-LVO was identified by follow-up vascular imaging at 5–7 days*.

b *This study included only patients treated with stent retrievers*.

c *This study included only patients treated with stent retrievers as the first-line treatment modality*.

d *This study included only patients treated primarily with mechanical thrombectomy*.

*TOAST, Trial of ORG 10172 in Acute Stroke Treatment; DSA, digital subtraction angiography; CTA, computed tomography angiography*.

Importantly, ICAS-LVO is considered a principal reason for failure of modern endovascular thrombectomy (17, 21, 23, 29). With modern endovascular modalities (e.g., stent retriever, contact aspiration thrombectomy), successful recanalization was possible in <30% of cases of ICAS-LVO (17, 23). Thus, the feasibility and safety of rescue endovascular modalities appropriate to ICAS-LVO (e.g., balloon angioplasty, stenting, intra-arterial glycoprotein IIb/IIIa inhibitor infusion) are constant points of discussion (17, 20, 23, 26, 28, 30–36). Although there is a lack of prospective studies regarding treatment of ICAS-LVO, most reports have indicated that ICAS-specific endovascular modalities are feasible in an acute setting. With optimal use of ICAS-specific endovascular modalities, patient outcome was also comparable to that of embolic occlusion (16, 23, 28). Finally, the rapid introduction of ICAS-specific endovascular modalities can be very important for timely and successful recanalization.

Therefore, determination of ICAS-LVO seems essential and critical for selecting an optimal endovascular treatment strategy. However, disappointingly, there is no corroborant method to identify ICAS-LVO. Such an identification method should be reliable and show acceptable sensitivity and specificity. It should be predictive of procedural details and applicable during or before EVT to help operators establish an optimal endovascular strategy. Some demographics, clinical risk factors, and imaging findings have been reported to be associated with ICAS-LVO (24, 32, 37–39). However, these demographics and clinical risk factors might not be specific to ICAS-LVO. Only atrial fibrillation proved to be fairly predictive for endovascular outcomes and is a clinically used risk factor (18, 24, 40). Although atrial fibrillation is associated with a higher probability of embolic occlusion, this association might be circumstantial (32). Certain imaging findings, such as a hyperdense artery sign or blooming artifact, might be helpful but are still controversial regarding role in the etiology of acute LVO (37, 41).

Unlike those less-specific identification methods, ICAS-LVO can be precisely identified angiographically (17, 32). Because angiographical determination has been most widely used in studies of ICAS-LVO, it is necessary to understand this method in depth to develop optimal endovascular treatment strategies. Among them, this review will discuss about the occlusion type—one of methods to identify the ICAS-LVO based exclusively on angiographical findings.

## Occlusion Type

Occlusion type is one of the most reliable angiographical surrogate markers for ICAS-LVO. Occlusion type was originally devised to differentiate ICAS-LVO from an embolic occlusion and was aimed for practical use in clinical settings (17). Before the introduction of occlusion type, clinical, and radiological findings (e.g., atrial fibrillation and hyperdense artery sign) were considered to presume an embolic occlusion. Although the concept of FFS was considered for similar purposes, it was used as merely an operational definition for ICAS-LVO and was not easy to apply during the procedure. More importantly, the predictive value of FFS for modern mechanical thrombectomy outcome has not been systemically evaluated (23). In contrast, occlusion type on computed tomography angiography (CTA) or digital subtraction angiography (DSA) was well correlated with procedural outcomes, especially in stent retriever thrombectomy (17, 18).

### Significance of Occlusion Type

The theoretical background of occlusion type for identification of ICAS-LVO is intuitive. For embolic occlusion, it is not likely for an embolus to be spontaneously halted in the middle of a normal artery. Instead, the embolus would likely become lodged at the site of an arterial bifurcation (i.e., a branching-site occlusion, BSO). In another words, an arterial occlusion found at the middle of an artery (i.e., truncal-type occlusion, TTO) is likely not caused by an embolus. In the narrow spectrum of occlusion etiologies of LVO, the TTO might be from an *in situ* thromboocclusion caused by an underlying ICAS.

According to a study that evaluated this hypothesis, TTO was significantly associated with stent retriever failure (odds ratio (OR): 32.2; 95% confidence interval (CI): 7.78–133.0) and with none of the embolic sources, such as cardioembolism and artery-to-artery embolism (OR: 9.07; 95% CI: 3.74–22.0) (17). Impressively, patients with a TTO showed a much higher rate of reocclusion events than those with a BSO (77.3 vs. 5.0%; *p* < 0.001). Furthermore, most patients (78.9%) eventually needed rescue modalities to achieve a successful recanalization. It seems evident that the clinical and endovascular details of TTOs are comparable to those of ICAS-LVO (16, 21, 23, 28, 29). Thus, in situations where no confirmative identification method for ICAS-LVO is feasible during the procedure, occlusion type could be a helpful surrogate marker to identify ICAS-LVO.

The ultimate goal in determining occlusion type was to help set up an optimal endovascular treatment strategy for ICAS-LVO. Therefore, the previous study originally focused on the predictive value of occlusion type for success of stent retrievers and the necessity of rescue modalities specific to ICAS-LVO. Based on the predictability of stent retriever successfulness in occlusion type, one could change endovascular modality from stent retriever to other modalities specific to ICAS-LVO earlier if TTO is observed. This strategy might avoid unnecessary trials of stent retriever and shorten procedural time. As for its practical application, occlusion type can be easily determined during endovascular procedures. Especially, in stent retriever thrombectomy, the occlusion type can be determined by a single stent retriever deployment across the occluded segment, as described in detail in the next section. Furthermore, occlusion type can be reliably determined before the endovascular procedure using preprocedural CTA (18). Early determination of occlusion type might be helpful in setting up the optimal endovascular treatment strategy. In addition, the predictive value of CTA-determined occlusion type was superior to atrial fibrillation or presence of a hyperdense artery sign, which have previously been the most widely considered pre-procedural identification methods, to presume occlusion etiology.

Clinical outcomes according to occlusion type was reported (42). In the single center report of 318 patients, the TTO group showed a comparable recanalization rate with the BSO group (80.4 vs. 88.5%; *p* = 0.097), although procedural details were completely different. With the comparable recanalization rate, clinical outcomes including favorable outcome (modified Rankin Scale score at 3 months 0–2; 46.4 vs. 46.9%; *p* = 0.944), symptomatic intracranial hemorrhage, and mortality were not significantly different between the TTO and the BSO groups.

### Determination of Occlusion Type During or Before Endovascular Procedures

**Figure 1:**
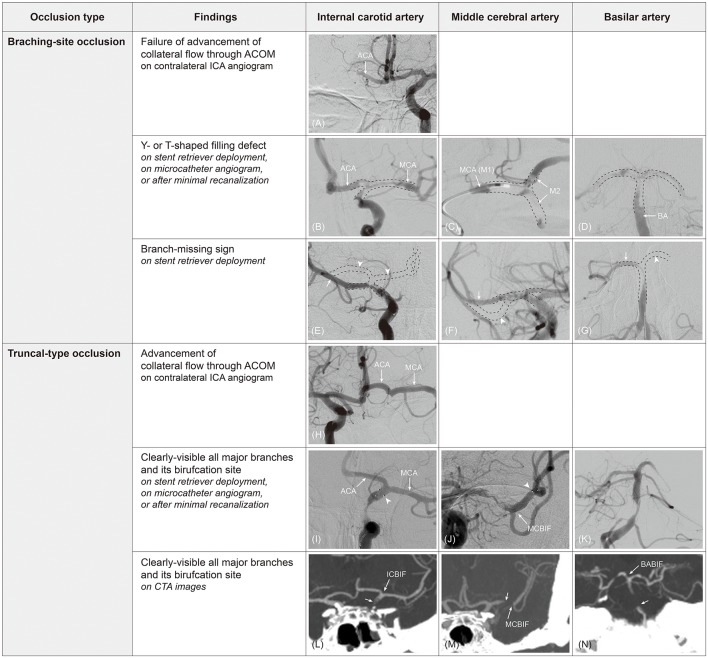
Determination of occlusion type by digital subtraction angiography (DSA) and computed tomography angiography (CTA). On contralateral internal carotid artery (ICA) angiogram, collateral flow cannot advance to ipsilateral middle cerebral artery (MCA) through anterior communicating artery (ACOM) because ipsilateral ICA bifurcation (ICBIF) site is involved in branching-site occlusion **(A)**. On the contrary, collateral flow can progress to ipsilateral MCA in truncal-type occlusion **(H)**. In branching-site occlusion, Y- or T-shaped filling defect (clot) involving arterial bifurcation site can be observed on stent retriever deployment **(B)**, on microcatheter angiogram **(C)**, or after minimal recanalization **(D)**. In addition, owing to the involvement of arterial bifurcation site, only one branch that stent retriever is deployed to can be seen on post-deployment angiogram in branching-site occlusion [arrow in **(E–G)**], while another branch is not seen [missing branch sign; arrowhead in **(E–G)**]. In truncal-type occlusion, all major branches and its bifurcation site can be clearly observed by stent retriever deployment [**(I,J)**; arrowhead, distal markers of stent retriever], on microcatheter angiogram beyond occlusion, or after minimal recanalization **(K)**. Those can also be observed on CTA images [**(L–N)**; arrow, original occlusion point]. ACA, anterior cerebral artery; M2, superior and inferior divisions of middle cerebral artery; MCBIF, middle cerebral artery bifurcation site; BABIF, basilar artery bifurcation site.

Occlusion type can be classified as either BSO or TTO during endovascular procedures. For angiographical determination of BSO, the following findings can be considered (Table 2). First, on contralateral internal carotid artery (ICA) angiography, collateral flow through the anterior communicating artery (ACOM) cannot advance to the ipsilateral middle cerebral artery (MCA) if the ipsilateral ICA bifurcation site is involved, a so-called ICA T-occlusion (BSO; Figure 1A). In contrast to that, the collateral flow can advance further to the ipsilateral MCA through the ACOM system if an occlusion is located below the bifurcation site (TTO; Figure 1H). This finding has been commonly observed at the start of endovascular procedures during collateral evaluation. However, with the recent push to shorten the time to recanalization, the target vessel is treated without collateral assessment in most current endovascular procedures.

**Table 2:** Angiographical findings suggestive of branching-site and truncal-type occlusions on catheter or digital subtraction angiography (DSA) and computed tomography angiography (CTA).

	**Branching-site occlusion**	**Truncal-type occlusion**
Principle	Involvement of the bifurcation site	Intact bifurcation site and all distal major branches beyond occlusion
DSA	(1) Failure of advancement of ACOM collateral flow to ipsilateral MCA^a^ (T-occlusion) (2) Direct visualization of Y- or T-shaped filling defect^b^^,^ ^c^^,^ ^d^ (Y- or T-shaped clot) (3) Absence or partial visualization of another branch^d^ (branch-missing sign)	(1) Advancement of ACOM collateral flow to ipsilateral MCA^a^ (below T-occlusion) (2) Clearly-visible distal major branches and bifurcation site beyond occlusion^b^^,^ ^c^^,^ ^d^
CTA	Vague or invisible major distal branches and bifurcation site beyond occlusion	Clearly-visible distal major branches and bifurcation site beyond occlusion

a *By contralateral ICA angiogram*.

b *By any angiography, including microcatheter angiography*.

c *By minimal or partial recanalization using a thrombectomy procedure*.

d *By post-deployment angiogram with stent-through flow*.

*ICA, internal carotid artery; ACOM, anterior communicating artery; MCA, middle cerebral artery*.

Second, Y- or T-shaped filling defects involving the bifurcation site can be directly observed during endovascular procedures by microcatheter angiogram, after partial recanalization by minimal thrombectomy procedure, or by angiogram performed with stent retriever in deployment (Figures 1B–D). Without doubt, these findings should be considered BSOs.

**Figure 2:**
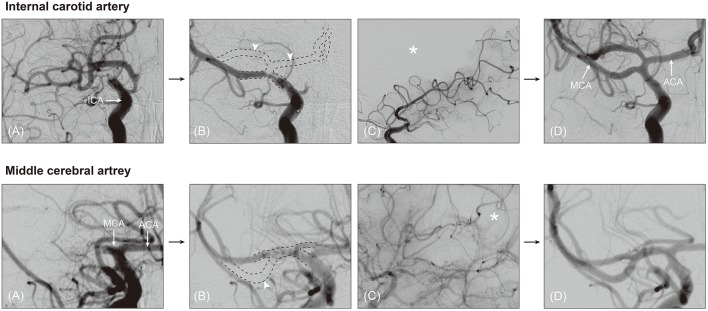
Angiographical territorial filling defect, suggestive of branching-site occlusion. On post-deployment angiogram during stent retriever thrombectomy, branch-missing sign can be observed (**A,B**, arrowhead). In patients whose missing branch is vague, angiographical filling defect could be helpful to guess the presence of the missing branch. On lateral view of post-deployment angiogram, corresponding territorial filling defect is seen (asterisk in **C**). On final angiogram, the missing branch is obviously observed **(D)**. ICA, internal carotid artery; MCA, middle cerebral artery; ACA, anterior cerebral artery.

Third, post-deployment angiography during stent retriever thrombectomy could give useful hints as to the occlusion type. For embolic occlusions in which an embolus might locate at the bifurcation site, the stent-through blood might only flow into the one branch where the stent retriever is deployed. Consequently, post-deployment angiography shows only one of all of the major branches (i.e., branch-missing sign, implicating BSO; Figures 1E–G). An angiographical territorial filling defect could be an indirect finding for the absence of the other major branches (Figure 2). If an occlusion is located at the arterial trunk, all major distal branches can be clearly seen by stent-through blood flow (TTO; Figures 1I,J).

To define the TTO, one should confirm that a bifurcation site and all its major distal branches are intact (distal confirmation). This distal confirmation can be performed by evaluating collateral flow through the ACOM system, microcatheter angiography beyond the occlusion site, stent-through blood flow across the occlusion site, or achievement of minimal recanalization (Figure 1K). More importantly, distal confirmation is also possible by CTA, which is more intuitive and easier than using catheter angiography or DSA (Table 2 and Figures 1L–N). In fact, its interrater agreement for classifying occlusion type was higher than that of DSA (kappa value 0.96 vs. 0.89) (18).

### Concerns With Regard to Occlusion Type

#### Disadvantages and Advantages in Determining Occlusion Type by DSA

Occlusion type is practically significant, is informative to set up an endovascular strategy, and is simpler to apply than FFS. In spite of these advantages, using DSA-determined occlusion type for identifying ICAS-LVOs has a few limitations. Above all, some additional manipulations are required for determination of occlusion type—for example, contralateral ICA angiography, microcatheter angiography beyond the occlusion, or post-deployment angiography. Among them, most determination practically depends on stent-through blood flow. Therefore, occlusion type often cannot be determined by DSA in patients who undergo non-stent retriever thrombectomy (e.g., contact aspiration thrombectomy), who did not have post-deployment angiography, or in whom stent-through blood flow was not achieved. In practice, occlusion type could not be determined by DSA in about 3.8% of patients (18). However, CTA findings are still helpful in those cases whose occlusion type cannot be determined by DSA.

One of most important advantages of occlusion type is that it is not affected by treatment results. In contrast with FFS, occlusion type can be determined even in cases of persistent occlusion or incomplete recanalization. Furthermore, occlusion type is obviously unaffected by the remnants of emboli, vasospasm, and iatrogenic artery dissection.

#### False Positives in TTO Determination by CTA (CTA-TTO With DSA-BSO)

Although CTA can be useful for identifying occlusion type, there is still the problem of false classification. DSA- and CTA-determined occlusion types can contradict each other. CTA-determined occlusion type was not in agreement with DSA-determined occlusion type in about 7.0% of patients (Table 3) (18). Among them, quite a few cases (62.5%, 10 of 16) had a DSA-determined BSO that was originally classified as a TTO on preprocedural CTA. One possible mechanism for this change in occlusion type is that distal migration of the clot which halted in the arterial trunk. In fact, half of these patients had a huge clot in the arterial trunk on DSA, which was observed as an angiographical filling defect (Table 4). Their original occlusion might have been a TTO in which the clot halted in the arterial trunk. However, on DSA, in addition to these clots in the arterial trunk, a distal BSO was also newly found. This finding is suggestive of distal migration of a proximal clot that halted in the arterial trunk to its distal bifurcation site, which finally led to the formation of a new, distal BSO that was not seen on preprocedural CTA. Similarly, in patient with BA occlusion, a new distal BSO might develop as a result of an artery-to-artery embolism from an ICAS-related lesion in the BA trunk. New distal BSOs were completely recanalized by stent retriever without residual stenosis in almost all cases. Although a few patients did not have a “truncal embolus” on DSA, complete distal migration of a proximal clot is a possible mechanism, in the same manner as described above.

**Table 3:** Consistency of CTA- and DSA-determined occlusion type.

	**CTA-determined occlusion type**	
	**Branching-site occlusion**	**Truncal-type occlusion**
DSA-determined occlusion type		
Branching-site occlusion	182 (96.8%)	10 (24.4%)
Truncal-type occlusion	6 (3.2%)	31 (75.6%)
Total	188	41

*This table was remade using the same study population in Baek et al. (18)*.

*CTA, computed tomography angiography; DSA, catheter or digital subtraction angiography*.

**Table 4:** Summary of 10 patients who had CTA-determined truncal-type occlusion and DSA-determined branching-site occlusion (CTA-TTO with DSA-BSO).

	**Occlusion site**	**AF**	**Reocclusion**	**mTICI**	**AOL**	**Recanalization modality**	**DSA findings suggestive of initial TTO**
1	ICA	+	–	2a	3	Stent retriever	None
2	MCA	–	–	2b	3	Stent retriever	None
3	ICA	+	–	2b	3	Stent retriever	Huge clot halted in cervical ICA
4	ICA	+	–	3	3	Stent retriever	Huge clot halted in cervical ICA
5	ICA	+	–	0	0	N/A	Huge clot halted in cervical ICA
6	ICA	+	–	0	0	N/A	None
7	ICA	–	–	2b	3	Stent retriever	Huge clot halted in cervical ICA
8	ICA	+	–	3	3	Stent retriever	Huge clot halted in cervical ICA
9	BA	–	+	3	3	Stent retriever for distal BA; PTA with stenting and GPI for BA trunk	Fixed focal stenosis in the BA trunk (as a tandem lesion)
10	ICA	+	–	2b	3	Stent retriever	None

*CTA, computed tomography angiography; DSA, catheter or digital subtraction angiography; AF, atrial fibrillation; mTICI, modified thrombolysis in cerebral infarction; AOL, arterial occlusive lesion; ICA, internal carotid artery; MCA, middle cerebral artery; BA, basilar artery; PTA, percutaneous transluminal angioplasty; GPI, glycoprotein IIb/IIIa inhibitor*.

It remains unclear why an embolus would halt in the middle of an artery. In those patients, the arteries did not show any morphological abnormalities that would explain why an embolus could be caught. Instead, we hypothesize that it might relate to the size of the embolus—a very large embolus might become compacted and ultimately lodged in the tortuous but not stenotic middle trunk of an artery (e.g., cavernous segment of internal carotid artery). Also, hemodynamic flow competition forces can affect the migration of emboli. For example, if a patient had sufficiently strong collateral flow through the ACOM to elicit effective retrograde flow, it might be possible for an embolus to not advance to its bifurcation site. In fact, in these patients, most truncal emboli were found in the cervical or petro-cavernous segment of ICA, in whom ACOM is quite thick and therefore cross collateral flows are sufficiently strong (Table 4).

#### False Negatives in TTO Determination by CTA (CTA-BSO With DSA-TTO)

Distal confirmation in CTA is dependent on visualization of a distal part of the artery beyond the occlusion. Because contrast media should reach the distal part of the artery beyond the occlusion, collateral flow is important in determining occlusion type by CTA. Collateral flow through the communicating arteries is important for distal confirmation in ICA or basilar artery occlusion. On the contrary, in MCA occlusion, leptomeningeal collateral flow, which shows greater individual differences, is important. Thus, CTA can misclassify truncal-type MCA occlusion as BSO if a patient has poor leptomeningeal collaterals to the MCA area. In fact, about 3.2% of CTA-determined BSOs were actually found to be TTOs on DSA (Table 3). Expectedly, all of these cases were MCA occlusions.

Given this, CTA-determined occlusion type, which showed a higher sensitivity for detecting the BSO compared to TTO, might be slightly biased toward identifying BSO. This limitation could be overcome by using multiphase CTA, which can allow for sufficient time for retrograde filling of contrast media into the distal part of the artery.

## Conclusions

Among only a few identification methods, the ICAS-LVO can be feasibly identified by angiographical findings. The identification of ICAS-LVO based on based on occlusion type, is a reliable and practical identification method for ICAS-LVO. Procedural details by occlusion type and its predictability to endovascular results were reported. Furthermore, occlusion type can be determined before or in the early stages of the procedure, which may be most helpful in setting up an optimal endovascular treatment strategy.

## Author Contributions

J-HB established the study idea, designed the manuscript structure, acquired and analyzed the data, and wrote the manuscript. BMK established the study idea, designed the manuscript structure, and made critical revisions to the manuscript with substantive intellectual content.

### Conflict of Interest Statement

The authors declare that the research was conducted in the absence of any commercial or financial relationships that could be construed as a potential conflict of interest.

## References

[1] Powers WJ Derdeyn CP Biller J Coffey CS Hoh BL Jauch EC. American heart association/american stroke association focused update of the 2013 guidelines for the early management of patients with acute ischemic stroke regarding endovascular treatment: a guideline for healthcare professionals from the american heart association/american stroke association. Stroke. (2015) 46:3020–35. 10.1161/STR.0000000000000074 26123479

[2] Powers WJ Rabinstein AA Ackerson T Adeoye OM Bambakidis NC Becker K. Guidelines for the early management of patients with acute ischemic stroke: a guideline for healthcare professionals from the american heart association/american stroke association. Stroke. (2018) 49:e46–110. 10.1161/STR.0000000000000158 29367334

[3] Hong KS Ko SB Yu KH Jung C Park SQ Kim BM. Update of the Korean clinical practice guidelines for endovascular recanalization therapy in patients with acute ischemic stroke. J Stroke. (2016) 18:102–13. 10.5853/jos.2015.01655 26846761 PMC4747068

[4] Linfante I Cipolla MJ. Improving reperfusion therapies in the era of mechanical thrombectomy. Trans Stroke Res. (2016) 7:294–302. 10.1007/s12975-016-0469-3 27221511 PMC4929023

[5] Hong KS Ko SB Lee JS Yu KH Rha JH. Endovascular recanalization therapy in acute ischemic stroke: updated meta-analysis of randomized controlled trials. J Stroke. (2015) 17:268–81. 10.5853/jos.2015.17.3.268 26437993 PMC4635708

[6] Lapergue B Blanc R Gory B Labreuche J Duhamel A Marnat G. Effect of endovascular contact aspiration vs. stent retriever on revascularization in patients with acute ischemic stroke and large vessel occlusion: the ASTER randomized Clinical trial. JAMA. (2017) 318:443–52. 10.1001/jama.2017.9644 28763550 PMC5817613

[7] Linfante I Starosciak AK Walker GR Dabus G Castonguay AC Gupta R. Predictors of poor outcome despite recanalization: a multiple regression analysis of the NASA registry. J Neurointerv Surg. (2016) 8:224–9. 10.1136/neurintsurg-2014-011525 25564538

[8] Song D Cho AH. Previous and recent evidence of endovascular therapy in acute ischemic stroke. Neurointervention. (2015) 10:51–9. 10.5469/neuroint.2015.10.2.51 26389007 PMC4571554

[9] Yoo AJ Andersson T. Thrombectomy in acute ischemic stroke: challenges to procedural success. J Stroke. (2017) 19:121–30. 10.5853/jos.2017.00752 28592779 PMC5466290

[10] Campbell BC Hill MD Rubiera M Menon BK Demchuk A Donnan GA. Safety and efficacy of solitaire stent thrombectomy: individual patient data meta-analysis of randomized trials. Stroke. (2016) 47:798–806. 10.1161/STROKEAHA.115.012360 26888532 PMC4760381

[11] Goyal M Menon BK van Zwam WH Dippel DW Mitchell PJ Demchuk AM. Endovascular thrombectomy after large-vessel ischaemic stroke: a meta-analysis of individual patient data from five randomised trials. Lancet. (2016) 387:1723–31. 10.1016/S0140-6736(16)00163-X 26898852

[12] Goyal M Jadhav AP Bonafe A Diener H Mendes Pereira V Levy E. Analysis of workflow and time to treatment and the effects on outcome in endovascular treatment of acute ischemic stroke: results from the SWIFT PRIME randomized controlled trial. Radiology. (2016) 279:888–97. 10.1148/radiol.2016160204 27092472

[13] Menon BK Sajobi TT Zhang Y Rempel JL Shuaib A Thornton J. Analysis of workflow and time to treatment on thrombectomy outcome in the endovascular treatment for small core and proximal occlusion ischemic stroke (ESCAPE) randomized, controlled trial. Circulation. (2016) 133:2279–86. 10.1161/CIRCULATIONAHA.115.019983 27076599

[14] Saver JL Goyal M van der Lugt A Menon BK Majoie CB Dippel DW. Time to treatment with endovascular thrombectomy and outcomes from ischemic stroke: a meta-analysis. JAMA. (2016) 316:1279–88. 10.1001/jama.2016.13647 27673305

[15] Bang OY. Considerations when subtyping ischemic stroke in Asian patients. J Clin Neurol. (2016) 12:129–36. 10.3988/jcn.2016.12.2.129 26833987 PMC4828557

[16] Al Kasab S Almadidy Z Spiotta AM Turk AS Chaudry MI Hungerford JP. Endovascular treatment for AIS with underlying ICAD. J Neurointerv Surg. (2017) 9:948–51. 10.1136/neurintsurg-2016-012529 27502403

[17] Baek JH Kim BM Kim DJ Heo JH Nam HS Song D. Importance of truncal-type occlusion in stentriever-based thrombectomy for acute stroke. Neurology. (2016) 87:1542–50. 10.1212/WNL.0000000000003202 27629085

[18] Baek JH Kim BM Yoo J Nam HS Kim YD Kim DJ. Predictive value of computed tomography angiography-determined occlusion type in stent retriever thrombectomy. Stroke. (2017) 48:2746–52. 10.1161/STROKEAHA.117.018096 28864601

[19] Gascou G Lobotesis K Machi P Maldonado I Vendrell JF Riquelme C. Stent retrievers in acute ischemic stroke: complications and failures during the perioperative period. AJNR Am J Neuroradiol. (2014) 35:734–40. 10.3174/ajnr.A3746 24157734 PMC7965801

[20] Jia B Feng L Liebeskind DS Huo X Gao F Ma N. Mechanical thrombectomy and rescue therapy for intracranial large artery occlusion with underlying atherosclerosis. J Neurointerv Surg. (2018) 10:746–50. 10.1136/neurintsurg-2017-013489 29203731

[21] Kang DH Kim YW Hwang YH Park SP Kim YS Baik SK. Instant reocclusion following mechanical thrombectomy of *in situ* thromboocclusion and the role of low-dose intra-arterial tirofiban. Cerebrovasc Dis. (2014) 37:350–5. 10.1159/000362435 24941966

[22] Kim YW Hong JM Park DG Choi JW Kang DH Kim YS. Effect of intracranial atherosclerotic disease on endovascular treatment for patients with acute vertebrobasilar occlusion. AJNR Am J Neuroradiol. (2016) 37:2072–8. 10.3174/ajnr.A4844 27313131 PMC7963784

[23] Lee JS Hong JM Lee KS Suh HI Choi JW Kim SY. Primary stent retrieval for acute intracranial large artery occlusion due to atherosclerotic disease. J Stroke. (2016) 18:96–101. 10.5853/jos.2015.01347 26467196 PMC4747073

[24] Lee JS Hong JM Lee KS Suh HI Demchuk AM Hwang YH. Endovascular therapy of cerebral arterial occlusions: intracranial atherosclerosis versus embolism. J Stroke Cerebrovasc Dis. (2015) 24:2074–80. 10.1016/j.jstrokecerebrovasdis.2015.05.003 26163890

[25] Lee YY Yoon W Kim SK Baek BH Kim GS Kim JT. Acute basilar artery occlusion: differences in characteristics and outcomes after endovascular therapy between patients with and without underlying severe atherosclerotic stenosis. AJNR Am J Neuroradiol. (2017) 38:1600–4. 10.3174/ajnr.A5233 28546252 PMC7960422

[26] Yi TY Chen WH Wu YM Zhang MF Chen YH Wu ZZ. Special endovascular treatment for acute large artery occlusion resulting from atherosclerotic disease. World Neurosurg. (2017) 103:65–72. 10.1016/j.wneu.2017.03.108 28377257

[27] Yi TY Chen WH Wu YM Zhang MF Zhan AL Chen YH. Microcatheter “first-pass effect” predicts acute intracranial artery atherosclerotic disease-related occlusion. Neurosurgery. (2018). 10.1093/neuros/nyy183. [Epub ahead of print]. 29790969

[28] Yoon W Kim SK Park MS Kim BC Kang HK. Endovascular treatment and the outcomes of atherosclerotic intracranial stenosis in patients with hyperacute stroke. Neurosurgery. (2015) 76:680–6. 10.1227/NEU.0000000000000694 25988927

[29] Matias-Guiu JA Serna-Candel C Matias-Guiu J. Stroke etiology determines effectiveness of retrievable stents. J Neurointerv Surg. (2014) 6:e11. 10.1136/neurintsurg-2012-010395 22591732

[30] Kang DH Yoon W Kim SK Baek BH Lee YY Kim YW Endovascular treatment for emergent large vessel occlusion due to severe intracranial atherosclerotic stenosis. J Neurosurg. (2018) 22:1–8. 10.3171/2018.1.JNS172350 29932374

[31] Baek JH Kim BM Kim DJ Heo JH Nam HS Yoo J. Stenting as a rescue treatment after failure of mechanical thrombectomy for anterior circulation large artery occlusion. Stroke. (2016) 47:2360–3. 10.1161/STROKEAHA.116.014073 27444259

[32] Lee JS Hong JM Kim JS. Diagnostic and therapeutic strategies for acute intracranial atherosclerosis-related occlusions. J Stroke. (2017) 19:143–51. 10.5853/jos.2017.00626 28592778 PMC5466291

[33] Chang Y Kim BM Bang OY Baek JH Heo JH Nam HS. Rescue stenting for failed mechanical thrombectomy in acute ischemic stroke: a multicenter experience. Stroke. (2018) 49:958–64. 10.1161/STROKEAHA.117.020072 29581342

[34] Fiehler J. Failed thrombectomy in acute ischemic stroke: return of the stent? Stroke. (2018) 49:811–2. 10.1161/STROKEAHA.118.020541 29581338

[35] Kim BM. Causes and solutions of endovascular treatment failure. J Stroke. (2017) 19:131–42. 10.5853/jos.2017.00283 28592777 PMC5466284

[36] Yang D Lin M Wang S Wang H Hao Y Zi W. Primary angioplasty and stenting may be superior to thrombectomy for acute atherosclerotic large-artery occlusion. Interv Neuroradiol. (2018) 24:412–20. 10.1177/1591019918763380 29562864 PMC6050887

[37] Cho KH Yoon Y Sohn SI Kim JS. Susceptibility vessel signs on T2^*^-weighted gradient echo images in patients with cerebral atherosclerosis. Int J Stroke. (2014) 9:E32. 10.1111/ijs.12318 25231582

[38] Kim SK Baek BH Lee YY Yoon W. Clinical implications of CT hyperdense artery sign in patients with acute middle cerebral artery occlusion in the era of modern mechanical thrombectomy. J Neurol. (2017) 264:2450–6. 10.1007/s00415-017-8655-0 29075836

[39] Kim SK Yoon W Kim TS Kim HS Heo TW Park MS. Histologic analysis of retrieved clots in acute ischemic stroke: correlation with stroke etiology and gradient-echo MRI. AJNR Am J Neuroradiol. (2015) 36:1756–62. 10.3174/ajnr.A4402 26159515 PMC7968760

[40] Hwang YH Kim YW Kang DH Kim YS Liebeskind DS Impact of target arterial residual stenosis on outcome after endovascular revascularization. Stroke. (2016). 47:1850–7. 10.1161/STROKEAHA.116.013046 27174525 PMC4927379

[41] Suh HI Hong JM Lee KS Han M Choi JW Kim JS. Imaging predictors for atherosclerosis-related intracranial large artery occlusions in acute anterior circulation stroke. J Stroke. (2016) 18:352–4. 10.5853/jos.2016.00283 27488977 PMC5066434

[42] Baek JH Kim BM Heo JH Kim DJ Nam HS Kim YD. Outcomes of endovascular treatment for acute intracranial atherosclerosis–related large vessel occlusion. Stroke. (2018) 49:2699–705. 10.1161/STROKEAHA.118.022327 30355204

